# Category boundaries modulate memory in a place-cell-like manner

**DOI:** 10.1016/j.cub.2024.09.083

**Published:** 2024-10-25

**Authors:** Stephanie Theves, Theo A.J. Schäfer, Volker Reisner, William de Cothi, Caswell Barry

**Affiliations:** 1 Max Planck Institute for Human Cognitive and Brain Sciences, Stephanstraße 1A, 04103 Leipzig, Germany; 2 Max Planck Institute for Empirical Aesthetics, Grüneburgweg 14, 60322 Frankfurt, Germany; 3 University College London, Department of Cell and Developmental Biology, Gower Street, London WC1E 6BT, UK

## Abstract

Concepts describe how instances of the same kind are related, enabling the categorization and interpretation of new information.^1,2^ How concepts are represented is a longstanding question. Category boundaries have been considered defining features of concept representations, which can guide categorical inference,^3^ with fMRI evidence showing category-boundary signals in the hippocampus.^4,5^ The underlying neural mechanism remains unclear. The hippocampal-entorhinal system, known for its spatially tuned neurons that form cognitive maps of space,^6,7^ may support conceptual knowledge formation, with place cells encoding locations in conceptual space.^4,8–11^ Physical boundaries anchor spatial representations and boundary shifts affect place and grid fields,^12–16^ as well as human spatial memory,^17–19^ along manipulated dimensions. These place cell responses are likely driven by boundary vector cells, which respond to boundaries at specific allocentric distances and directions,^20–23^ the neural correlates of which have been identified in the subiculum and entorhinal cortex^20,24,25^. We hypothesize similar patterns of memory adaptations in response to shifting category boundaries. Our findings show that after category boundary shifts, participants’ memory for category exemplars distorts along the changed dimension, mirroring place field deformations. We demonstrate that the boundary vector cell model of place cell firing best accounts for these distortions compared with alternative geometric explanations. Our study highlights a role of category boundaries in human cognition and establishes a new complementary link between hippocampal coding properties with respect to boundaries and human concept representation, bridging spatial and conceptual domains.

## Results

**Figure 1:**
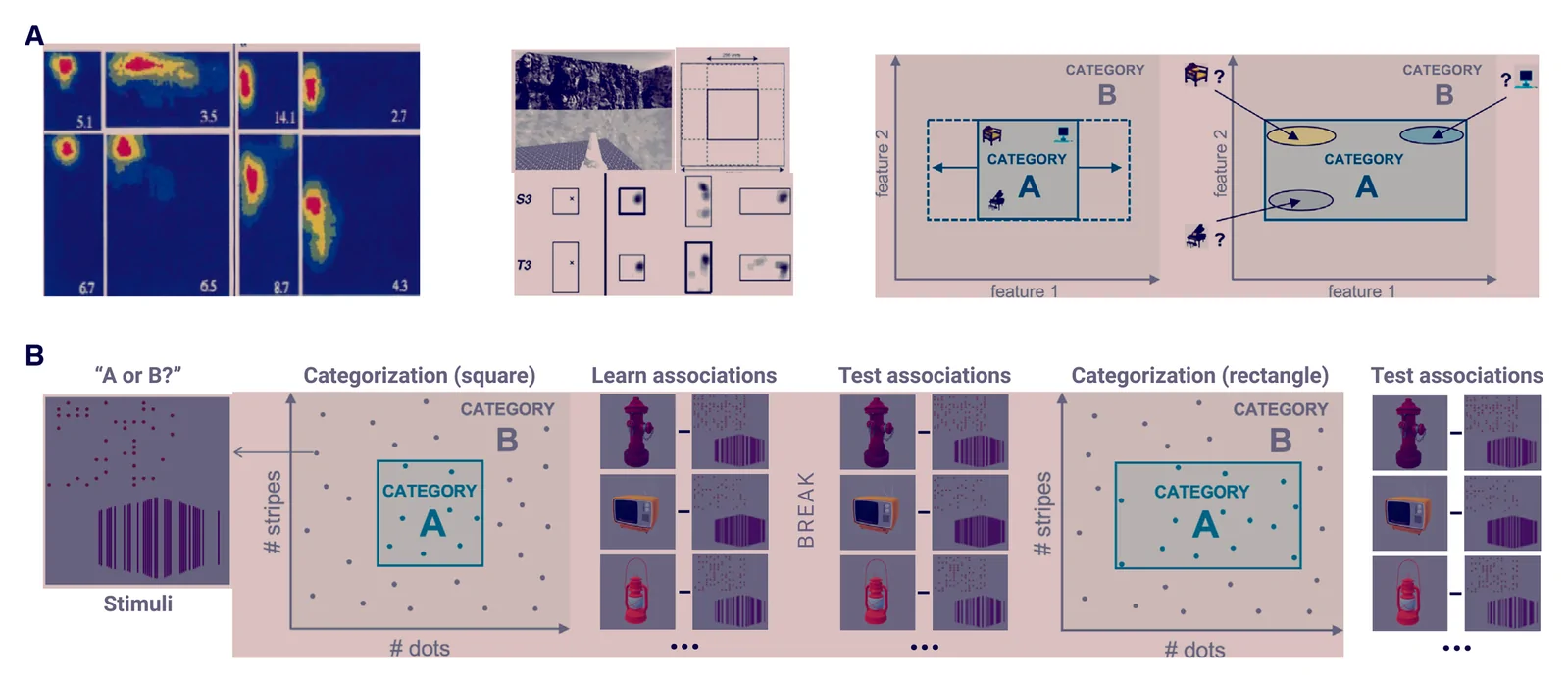
Background and experimental design (A) Spatial and conceptual boundary changes. Left: deformations of place fields in response to changes of environmental geometry. Depicted are firing fields of two place cells (delineated by the line) when the shape of a rodent’s recording box changes from a square (upper-left, respectively) to a rectangle or large square (from O’Keefe and Burgess^14^). Place fields systematically adapted along the changed dimension. Middle: congruent with the idea that place cells serve as the neural substrate for spatial memory^17^ demonstrated that corresponding boundary changes in a virtual arena produce similar distortions in human memory for object locations (from Hartley et al.^17^). Right: illustrates prediction of a category extension. Category boundaries shift to include new members of category A. If exemplar representations are sensitive to the geometry of the concept space, they might become distorted toward the new boundaries (e.g., memorize the chair with lower than actual feature 1 values). (B) Experiment (task sequence from left to right): participants were trained to categorize stimuli as A or B based on the combination of their dot- and stripe-frequencies (schematic illustration of feature space and boundaries), e.g., the depicted example stimulus with low dot- and high stripe-frequency belongs in category B. Subsequently, participants learned to associate 5 objects with 5 category A stimuli (see Figure 3 for the 5 object positions). In a second categorization task, boundaries were shifted. On which dimension the shift occurred was counterbalanced across participants. Before and after the shift, the object associations were tested by asking participants to generate the feature values for each object within the category A space. Related to Figure S1.

Based on the notion that the place cell system may encode conceptual knowledge, we tested whether memory of exemplars is distorted by shifting category boundaries in a similar manner as place cells and spatial memory by physical boundary changes (Figure 1A). Category boundaries may shift when updating a concept by new exemplars (e.g., from “flying” toward “flightless” animals when a new “flying” exemplar is heavier than previous category members). In our experiment (Figure 1B), participants learned to categorize stimuli into two categories, which were defined as a square enclosure (category A) and its surrounding area (category B) in a two-dimensional feature space. Five exemplars of category A were further associated with objects. In a second categorization task, participants encountered that the category boundary had shifted in one dimension, transforming category A into a rectangular shape. Critically, we tested participants’ memory of the five object-cued exemplars before and after the boundary shift.

### Category and association learning

**Figure 2:**
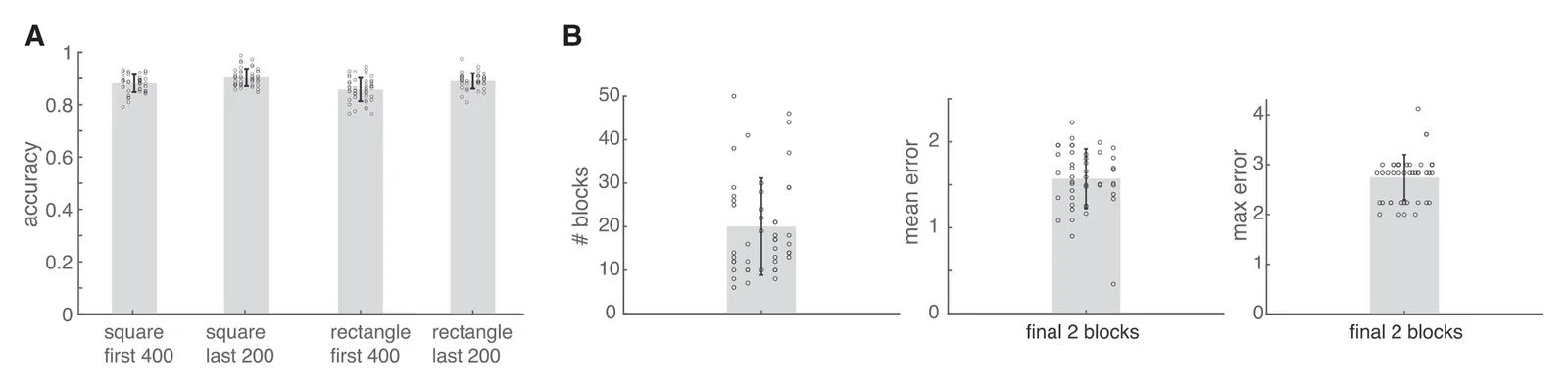
Training: Categorization and associative learning performance (A) Mean categorization accuracy for the first 400 trials and the final 200 trials is displayed for the first (square category A) and second (rectangular category A) categorization task. (B) Learning associations between objects and category A stimuli (feature combinations). Displayed are the number of blocks participants required to reach the criterion (left), the mean (middle), and the maximal displacement (right) in the last two blocks. Bars denote the mean, lines denote standard deviation, and circles denote individuals.

Participants learned and subsequently updated the concept of two categories (category A being a delineated from B via square/rectangular boundaries; Figure 1B) via categorization tasks. Stimuli varied in the frequency of dots and of stripes, and the combination of these values defined category membership. Participants responded to a single stimuli and received feedback whether the selection (A or B) was correct. After a minimum of 400 trials (accuracy square: 88.2% ± 3.3% [mean ± SD; *N* = 46; rectangle: 85.7% ± 4.4% [*N* = 46]; Figure 2A left), if accuracy did not reach 85% in each region of the feature space, training proceeded until the criterion was reached in the last 200 trials (average accuracy: square: 90.16% ± 3.34% [*N* = 45; one participant already reached the criterion in the first 400 trials]; rectangle: 89.09% ± 2.95% [*N* = 41; five participants already reached the criterion in the first 400 trials]; Figure 2A right). The final 50 test trials confirmed that participants learned the categorization rule well (square: 83.00 ± 6.89 [*N* = 46]; rectangle: 83.16 ± 7.38 [*N* = 43]; three participants did not perform this test). Subsequent to the first categorization task (square), participants learned to associate five objects with certain category A stimuli. To ensure precise encoding, participants were trained to generate the feature combinations (by upregulating and downregulating dot and stripe frequency) associated with each object until reaching a mean/maximal displacement of ≤2.5/3 steps in the last two blocks (number of blocks: 20 ± 11, 6–50 blocks [mean ± SD, range]; final performance: mean error: 1.571 ± 0.347, max error: 2.746 ± 0.454, [*N* = 46]; three participants who terminated training due to time constraints but close to meeting the criterion [max error: 3.6; 4; 3.6] were included in the sample; Figure 2B). In sum, the training data show that participants learned the categorization rule and the object associations well.

### Mnemonic distortions following category boundary changes

**Figure 3:**
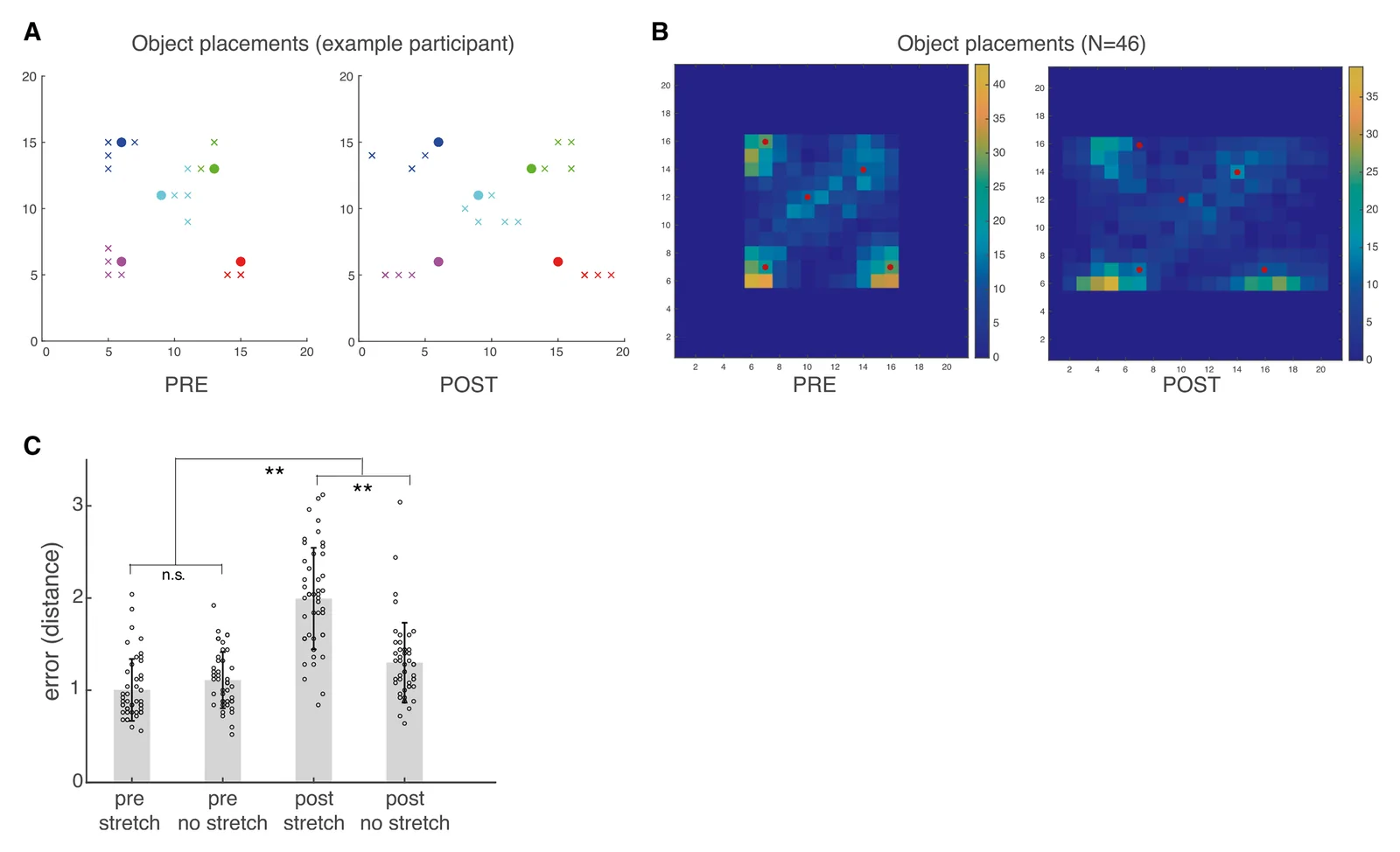
Test: Memory distortions following category boundary change Distribution of placements in the associative memory test before (pre) and after (post) recategorization. (A) Responses of a single participant (x denote responses; circles denote true position). (B) Response density map of all participants (dots denote true positions, color bar reflects total count, axes denote feature coordinates). (C) Displacements (Euclidean distance from true position) significantly increase from PRE to POST on the stretched as compared with the non-stretched dimension (bars reflect means, dots reflect individuals, asterisk denote significance at *p* = 0.001). Related to Figure S2.

We tested participants’ memory of the object-associated feature combinations before (pre) and after (post) the second categorization task, to evaluate whether the category boundary change distorted their memory of the cued exemplars. Participants had to reconstruct the feature combination of a cued object within the category A space. In the pre-test, memory accuracy was at a similar level as at the end of training (mean displacement: 1.72 ± 0.38; *N* = 46). The main interest was whether the memory of the cued exemplars is specifically distorted (i.e., deviates from the learned features) along the stretched as compared with the non-stretched dimension from pre-to post-category boundary change. The effects of time point (pre and post), dimension (stretched and non-stretched), and their interaction on displacements were evaluated in a two-factorial ANOVA. We found a main effect of time point (F(1, 180) = 91.91, *p* < 0.00001), a main effect of dimension (F(1, 180) = 22.88, *p* < 0.0000), and critically, a significant interaction effect (F(1, 180) = 42.34, *p* < 0.00001) on displacements (Figure 3C). In line with our hypothesis, post-hoc one-sample t tests revealed no significant differences in displacements between the stretched and non-stretched dimensions in the pre-test (t(45) = −1.759, *p* = 0.089) but significantly larger displacements in the stretched than the non-stretched dimension in the post-test (t(45) = 7, *p* < 0.0001; Bonferroni-adjusted alpha level of 0.025; replication of results in uncorrected trials in Figure S2). The test results indicate that mnemonic representations are sensitive to category boundaries.

### A BVC population account of concept updating

We find that participants’ memory for the feature combinations associated with the objects adapted to the new boundaries. Similar to place fields, memory was specifically distorted along the stretched as opposed to the non-stretched dimension. We next asked whether this pattern of memory distortions is best accounted for by a boundary vector cell (BVC) model of place cell population activity as compared with simple geometric accounts, which predict remembered positions in the new environment maintain a fixed distance (FD) to the nearest two walls or a fixed ratio (FR) to the opposing walls.^22^ The sensitivity of place cells to the environmental geometry is assumed to be driven by the integrated input of BVCs in the subiculum and entorhinal cortex, that fire with a preferred distance and allocentric direction to environmental boundaries.^14,20,25^

**Figure 4:**
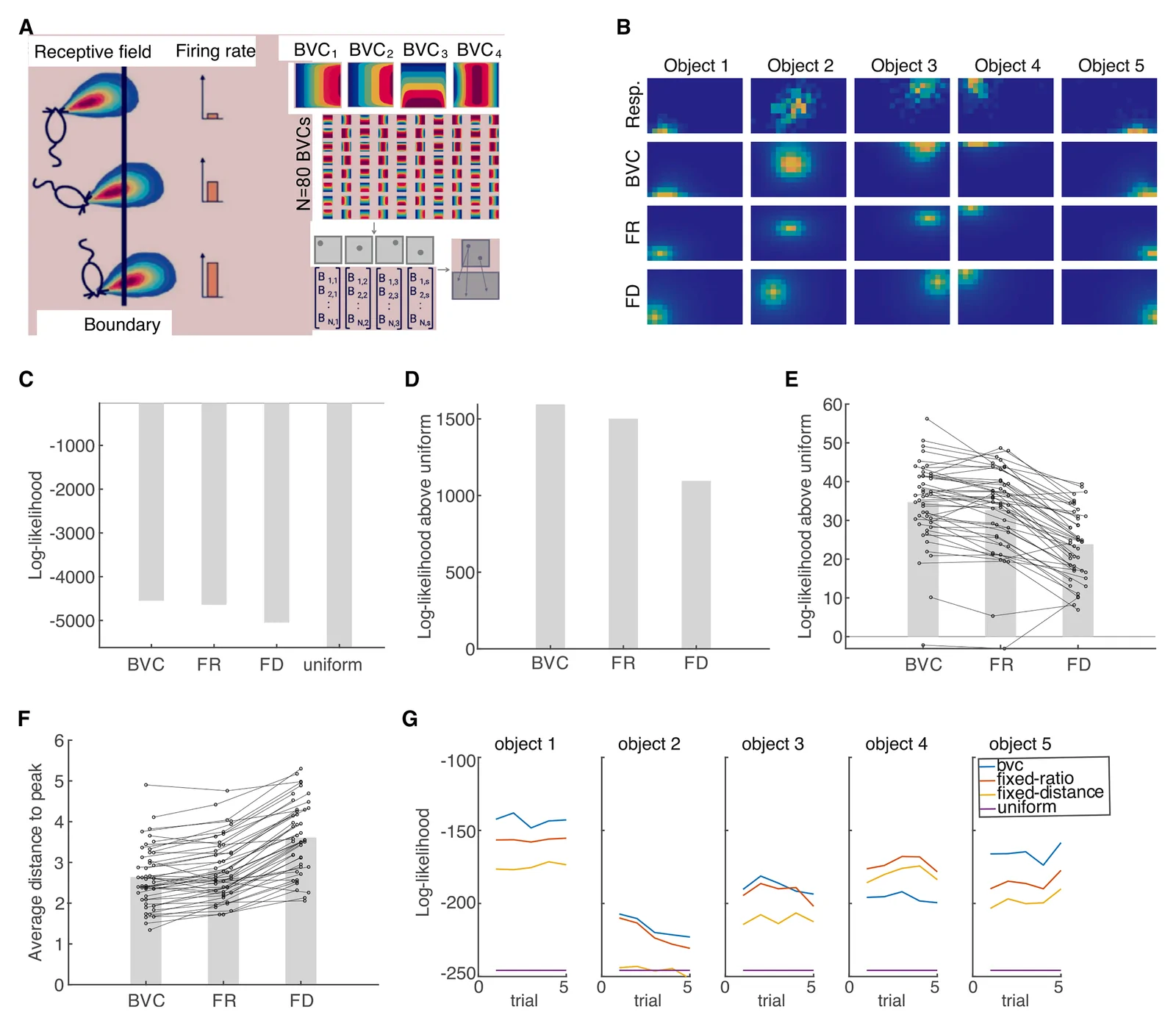
Modeling mnemonic distortions: Boundary vector cell model and displacement predictions (A) Boundary vector cells (BVCs) fire at a preferred distance and allocentric direction to walls (left; adapted from Lever et al.^25^). We simulated the population response of 80 BVCs for each location in each environment. Displacement predictions are based on the similarity between the response vectors for each location in the square and each location in the rectangle (right). (B) Response distribution of object placements in the POST memory test (first row) and displacement predictions of three models (second to last row: BVC, fixed distance [FD], fixed ratio [FR]). (C–E) The BVC model provides the overall best fit to the response distributions. Displayed are (C) the absolute loglikelihood and (D) the likelihood above a uniform distribution when the models are fit to all data or (E) to single participants. (F) Replacement distance from the peak predictions per participant. (G) The BVC model (blue) provides the best fit to four of five object locations as compared with FR, FD, and a uniform distribution. Bars denote the mean; dots denote single participants. Related to Figures S3 and S4.

In brief, we generated population responses for 80 BVCs with firing fields at different preferred distances and directions to walls for each location in the square and in the rectangular environment. Placement predictions were derived by comparing the similarity between the response vectors at each location in the rectangular environment with the object-specific response vectors from the square environment (Figure 4). We find that the BVC model provides the overall best fit to placement responses, followed by the FR and then the FD model. The BVC model consistently provided the most likely fit to the behavior, both when fitted across all participants (Figures 4C and 5D; log-likelihood (LL) above uniform; BVC: 1,594, FR: 1,501, FD: 1,096) and when fit to individual participants (Figure 4E; BVC: 34.67, FR: 32.64, FD: 23.83), as well as the smallest mean distance between displacements and peak prediction (Figure 4F; one-factorial ANOVA: F(2, 135) = 19.86, *p* < 0.0001; one-sample t test: BVC vs. FR: t(45) = −5.06, *p* < 0.0001; BVC vs. FD: t(45) = −11.7, *p* < 0.0001, Bonferroni-corrected alpha = 0.025). The BVC model provided the best fit to four out of five object locations, while the data for position 4 were best accounted for by the FR model (Figure 4G).

## Discussion

This study shows that updating concepts by shifting category boundaries can distort exemplar memory, and that the pattern of distortions resembles the effects of environmental boundary changes on place cell firing and spatial memory. We further demonstrate formally that the pattern of memory changes after category boundary shifts is predicted by a BVC model of place cell population activity, providing a new complementary link between concept formation and representational mechanisms arising from the tuning of hippocampal cells.

How concepts are represented is central to the study of human cognition. Cognitive theories related to generalization and categorization in particular posited the integration of multiple relations between experiences in a common representational space, with an appeal of vector space representations in capturing relational similarity in a domain general manner.^6,26,27^ A potential neural substrate for such representational formats might be provided by spatially tuned cells such as place cells,^7^ grid cells,^28^ and BVCs,^20,24,25^ which form cognitive maps of the environment.^7^

Recent studies have identified commonalities between spatial and non-spatial relation representations in the hippocampal system for both rodents and humans,^4,8–11,29–32^ including fMRI evidence for a hippocampal role in the formation and representation of knowledge structures, i.e., concepts, in category-learning tasks.^4,9,10,33–35^ Specifically, hippocampal activation patterns have been shown to reflect distances between exemplars as well as decision boundaries along category-defining dimensions.^4,9^ As a window into the potential neural mechanisms underlying concept learning and its shared foundation with spatial cognition, we compared the effects of category boundaries on memory with neurobiologically inspired predictions from a place cell model. We tested participants’ memory of category exemplars before and after updating the category boundaries in terms of a one-dimensional stretch of the initial category space. Memory of cued exemplars was specifically distorted along the stretched compared with the non-stretched dimension, mirroring known patterns of place field deformations. This distortion was not necessarily to be expected, as participants could just have recalled the absolute feature combination they had learned. The specificity of the effect to the stretched dimension further discounts the possibility that these changes are driven by general repulsion or memory dispersion effects over time. While previous work established influences of category boundaries and long-term semantic category structure on similarity judgements^36^ or new learning^37^ (i.e., relative location of items on a screen were better learned when congruent with their semantic similarity^37^), we believe that these results provide a new demonstration of how category boundary changes can shape memory.

The observed distortions were most accurately accounted for by a BVC model, which outperformed simpler geometric models that predicted replacements, either at FR to opposing boundaries or at FD to neighboring boundaries, equally for all cue locations. In contrast, the biological plausibility of the BVC model is underlined by its ability to account for the influence of a cue’s proximity to boundaries.^17^ Thus, our findings transfer and extend prior work on the influence of environmental geometry on place and grid cell firing^14,38^ and on human spatial memory^17,39^ to the non-spatial cognitive domain. Specifically, our model comparisons align with the results of Hartley et al.,^17^ which demonstrated that environmental boundary shifts in a virtual reality setting distort human memory for object locations in a manner akin to rodent place cell behavior. Likewise, Chen et al.^39^ showed that human self-location estimates display biases aligned with the rescaling of grid code observed in rodents. The parallelism between cell tuning effects and spatial memory outcomes suggests that both are influenced by similar underlying processes. Extending these findings to the realm of concept learning, our study posits that such correlations between cell tuning and behavior may also apply to higher-level cognitive tasks.

Our findings shed new light on the mechanism underlying concept learning by manipulating and evaluating the representation of category boundaries. Classic models differed in which feature of the representational space guides behavior: while prototype and exemplar models emphasize the center, decision bound theory suggests that category learning proceeds by developing bounds between category regions, which then serve as reference to interpret new exemplars.^3,40^ How well each account describes behavior depends on task properties^41^ and, in line with the involvement of multiple systems in concept learning,^42,43^ multiple formats may also coexist in different brain regions.^43,44^ A question in this context is whether and how the respective features are realized in neural processing, e.g., whether category boundaries are featured in neural concept representations. In fMRI studies, hippocampal activity tracked the distance to the category boundary during categorization^5^ as well as during passive viewing of exemplar-associates after categorization,^4^ whereby the latter representation is dissociated from task difficulty (which inversely relates to boundary distance). The present results support the idea that spatial cells in the hippocampal system may encode category boundaries in a similar manner to physical boundaries and may hint at a potential mechanism underlying the reported fMRI-based boundary signals.

The BVC model most accurately accounted for the impact of category boundaries on memory, except for object position 4 on which it performed less well. While this difference might simply be a matter of chance, it is interesting to note that positions 4 and 5 (on which the BVC model performs well) have a low/high combination of feature values but differ in whether the low feature value lies on the stretched (x) or non-stretched dimension (y). This difference might be important, as it is possible that lower feature values are more attended to or better encoded, making memory for those features less susceptible to the stretch manipulation. Supporting this, we observed lower pre-test memory errors for the x-feature compared with the y-feature at position 4, with the reverse trend for position 5 (Figure S3). The key point is that feature spaces, unlike physical spaces, have dimensions with qualitative differences that could interact with boundary-dependent memory adaptation in ways that the BVC model does not capture. Therefore, the interaction between potential boundary-sensitive codes and domain-specific mechanisms remains an area for future research.

In this experiment, the suspected mechanism becomes visible through erroneous behavior (memory distortions), which may spark consideration of its cognitive relevance. Similar to fMRI studies on hippocampal contributions to concept learning, we evaluated the early stage of concept formation. In the early stage, when updates occur frequently, adjusting formed maps (i.e., population code of grid-and place cells) to cover moderate variations of the original space, can provide a dynamic and flexible approximation. Thus, the coding regime of the hippocampal-entorhinal system may afford rapid formation and dynamic updating of new concepts at the cost of being accompanied by transient imprecision. In the spatial domain, boundaries comparably serve as cues for reorientation and are thought to anchor and update spatial representations.^45–50^ While the concept participants learned was defined along two perceptual feature dimensions, real-world concepts can be higher-dimensional and defining features derived from different content domains, including non-perceptual features. The design did not aim to explain the long-term representation of conceptual knowledge but to provide a methodological window into the mechanisms underlying its formation. As there is a limit to the number of new (uncorrelated) dimensions that can be considered at the same time, we consider the present findings as principally generalizable in this respect. Furthermore, the firing properties of BVCs and place cells are theoretically not restricted to two dimensions; in particular empirical evidence for place cells extends to 3D.^51^ Lastly, hippocampal and entorhinal cells have been shown to encode relationships in non-spatial domains^30^ and abstract task variables,^52,53^ and corresponding representational signatures have been observed via human fMRI including perceptual^31^ and non-perceptual abstract^29,32^ (e.g., social) dimensions.

Our approach builds on prior parallels between human memory responses and firing patterns of rodent place and grid cells in response to geometric deformations of the environment.^15,17,18,39^ We tested predictions derived from the similarity of modeled BVC population activity in the original and deformed space, as respective predictions have been shown to account for the pattern of place field and human spatial memory distortions in response to environmental geometry changes.^17^ Intracranial recording and neuroimaging studies of the human medial temporal lobe have further revealed neural tuning and populational-level representations of cognitive spaces paralleling those observed in rodents.^8,9,54–57^ A contribution of place field deformations to this category-boundary anchored memory shifts is therefore conceivable. Yet, alternative neural implementations of the observed behavior cannot be excluded. Given the qualitative differences between feature-based and spatial relations, future work might further explore the interaction of potential place field deformations and additional domain-specific mechanisms in generating the observed behavior.

In sum, this study offers insights in how category boundaries can shape memory and suggests a biological account in form of a place cell model. The results support the notion of shared representational mechanisms underlying spatial and non-spatial cognition and demonstrate a novel link between properties of hippocampal coding and concept learning. This might open up new avenues for further in-depth investigations of elementary neural mechanisms underlying higher-level cognition in humans.

## Resource Availability

### Lead contact

Further information and requests should be directed to the lead contact, Stephanie Theves (theves@cbs.mpg.de).

### Materials availability

This study did not generate new unique reagents.

### Data and code availability

Data reported in this paper and the code are reposited at Zenodo and are publicly available at https://doi.org/10.5281/zenodo.13969607 as of the date of publication.

## Star★Methods

### Key Resources Table

**Table:** 

REAGENT or RESOURCE	SOURCE	IDENTIFIER
Data and code	https://zenodo.org	https://doi.org/10.5281/zenodo.13969607

### Experimental Model and Study Participant Details

Sixty-eight participants gave written informed consent and were compensated for participation as agreed by the local Research Ethics Committee (Medical University Leipzig). Thirteen participants did either not reach the performance criteria of the training phase (see below; associative learning and categorization tasks) or did not finish the entire experiment. Nine participants had to be excluded due to an initial error in the task script. Thus, forty-six participants (age: 25.7 ± 3.6 years; 26 female) entered the main analysis to evaluate category boundary effects on memory.

### Method Details

#### Experimental procedure

Participants performed five behavioural tasks on the same day. In a first categorization task, they acquired the concept of two stimulus categories, which were defined as a square enclosure (category A) and its surrounding (category B) in a two-dimensional feature space. Participants learned to categorize stimuli with varying number of dots & stripes based on the combination of these features (1). Subsequently, they learned to associate five category-A stimuli with everyday objects (2). As the key manipulation, we then relocated the category boundaries, such that the shape of category A changed from a square to a rectangle (Figure 1). Participants encountered this change in a second recategorization task (4). We then probed participants memory of the stimuli associated with the objects before (3) and after (5) the recategorization task (i.e., they had to reconstruct the feature-combination associated with each object). As knowledge of the category boundaries and the associations was a precondition to test for effects of boundary updates on memory (3, 5), we set rather high performance criteria for the training tasks to inform inclusion in the test (approximated based on previous experience with these tasks^14,15^). As the experiment took several hours (including breaks) with variance among participants, participants that terminated a task due to time constraints or fatigue but performed close to the criterion were included.

#### Categorization task

Participants were presented one stimulus at a time and had to indicate via button press (response time limit: 10 s) whether it belongs to category A or B and subsequently received feedback (500 ms) whether the response was correct. Stimuli were grey squares with varying numbers of red dots and purple stripes in the lower and upper part of the grey square, respectively (example stimulus Figure 1 *left*). Category membership was determined by the combination of dot- and stripe-frequency. Specifically, the category boundary was defined by a square-shaped category A within a surrounding category B in a two-dimensional feature space (dimensions: dot- and stripe-frequency). The size of the feature-space was 21×21 steps in dot- and stripe-frequency (the size of the steps was adjusted using Maximum Likelihood Difference Scaling^58^ to achieve equal psychophysical discriminability; dimensions ranged between 16-190 dots and 6-64 stripes). The boundaries of category A changed from 5:15/5:15 (square) in the first categorization task to 1:19/5:15 (rectangle) in the second categorization task. Response button to category mapping was balanced across participants. The categorization phase proceeded as follows: Participants performed a minimum of 400 trials, in which A and B stimuli appeared with an equal probability (on average 317±3 (mean±std) different stimuli sampled). If participants did not reach 85% accuracy in all sectors of the feature space (evaluated for eight sectors: left and right to category A within the y-axis limit of A, above and below A within the x-axis limit of A, and accordingly four remaining corner spaces), then training continued for at least 200 trials with sampling adapted to sectors below 85% until the criterion was reached for the last 200 trials. To aid initial learning, in the experiment the four feature values that demark the boundaries (i.e., stimuli displaying only the stripes or only the dots at either the minimal or maximal frequency, respectively) were displayed in a horizontal arrangement at the bottom of the screen. In a final test this display was removed and participants performed 50 unique trials without repetition. The recategorization task (rectangular category A) was alike to the first categorization task (square category A) in all aspects except the category boundary values. Participants were informed that they perform the same task but that the categorization rule might have changed to some extent. The assignment of feature dimension (dots/stripes) to manipulation (stretched/non-stretched dimension) was counterbalanced across participants.

#### Associative learning task

The five associations of object and stimulus (dot-stripe feature combination) were randomized across participants. All five associations were presented once. In subsequent trials, participants had to construct the stimulus associated with a given object by up- and down-regulating the dot- and stripe-frequency of a random start stimulus via four adjacent keys (stripes up, stripes down, dots up, dots down). To avoid that participants consider specific perceptual patterns (rather than frequency), the stimuli were generated by randomly distributing dots and stripes within a given area of the stimulus. Accordingly, a certain position in concept space (e.g., co-ordinate 15(dots)/10(stripes)) never looked exactly the same, except that the number of dots and stripes was identical between two iterations of this position. Upon editing the stimulus associated to the object cue, participants confirmed their choice via button press. If the response was not correct, they were presented with the correct stimulus before being asked to repeat the trial until the correct combination was generated. The five associations (1 block) were iterated until participants reached an average deviation below 2.5 steps and a maximal deviation below 3 steps in the 21×21 steps sized feature space within the last 2 blocks.

#### Associative memory test

The associative memory test followed the same procedure as associative learning, except that upon confirmation of the generated stimulus, no feedback was provided. Participants performed five iterations of each association (in total 25 trials). The associative memory test was performed immediately before and after the recategorization task. All objects were positioned within the initial category A. In both tests, responses that were outside the initial or stretched category A respectively, had to be adjusted. This was done, because the model analysis required responses to be inside the boundaries. The overall number of corrected trials was relatively low (number of corrected trials in POST: 3,6 ± 2,7 trials, median: 3 trials). The effects of dimension (stretched/non-stretched) and time (pre / post-stretch) on displacement error were evaluated in a two-factorial ANOVA. As control analyses (Figure S2), the ANOVA was repeated using only trials in which participants received no correction, and when additionally excluding participants that received more than 2 corrections in the session to avoid general influences on behavior.

### Quantification and Statistical Analysis

We evaluated whether memory responses following category boundary changes are explained by a model of boundary vector cell (BVC) population activity. BVCs are cells in the subiculum that fire with a preferred distance and allocentric direction to a wall and are assumed to drive the geometry-sensitivity of place cells.^20,25^ We compare this model to two alternative geometric accounts, that predict that remembered positions in the rectangular environment maintain either a fixed distance to the nearest two walls (FD) or a fixed ratio (FR) to the opposing walls,^17^ respectively. For this purpose, we fitted three models to the distribution of displacements in the post-recategorization memory test.

#### Boundary vector cell model

We generated response vectors of a BVC population (N=80) for each location in a square (100 × 100 virtual units) and rectangular (100 × 173) environment (see Figure S4). Displacement predictions were derived by calculating the dissimilarity in the BVC response vectors across locations in the square and rectangle. Specifically, dissimilarity was calculated using the L2 norm between response vectors. First, BVC response vectors corresponding to cued object locations in the square environment were stored as a reference. Next, we calculated the dissimilarity (L2 norm) between these object-specific response vectors in the square environment, and each possible location in the rectangular environment. Locations defined in a square, have no ‘true’ position defined in a rectangular space. However, these dissimilarity scores can instead be used to map out which positions in the rectangular environments are most dissimilar to the cued object locations in the square. Dissimilarity scores were then turned into similarity scores by subtracting the dissimilarity from its maximum value across the rectangular environment, before down-sampling them onto the size of the concept spaces (square: 11×11 grid, rectangle: 11×19 gid). Thus, predicted responses cluster around locations that minimize the net change in activity from that seen at the cued locations in the square environment.

The population of BVCs were defined by a similar specification used in previous iterations of the BVC model.^22,38^ Specifically, a boundary that is at distance r, direction θ and subtending at an angle *δθ* contributes to the firing rate *f*_*i*_ of the i^th^ BVC is according to:

$$\delta f_{i}=g_{i}(r,\theta )\delta \theta$$

where:

$$g_{i}(r,\theta )\propto \frac{\exp\left[-{\left(\text{r}-{\text{d}}_{\text{i}}\right)}^{2}/2\sigma_{\text{dist}\;}^{2}\right]}{\sqrt{2\pi \sigma_{\text{dist}\;}^{2}}}\times \frac{\exp\left[-{\left(\theta -\phi_{\text{i}}\right)}^{2}/2\sigma_{\text{ang}\;}^{2}\right]}{\sqrt{2\pi \sigma_{\text{ang}\;}^{2}}}$$

Here, ϕ_i_ = [0°, 90°, 180°, 270°] are the BVC angular tunings and the 20 distal tunings were chosen to evenly span the stretched space e.g. *d*_*i*_ = 173 − 8:87*α* virtual units, for *α* taking integer values ranging from 0 to 19 (inclusive). These 4 angular and 20 distal tunings give rise to the N=80 BVCs defined above with σ_*dist*_ = 200 virtual units and σ_*ang*_ = 11.25°. This instantiation of the BVC model was adapted to the concept space implemented in this study by having preferred angular tunings defined by the axes of motion in the concept space (i.e. ϕ_i = [0°, 90°, 180°, 270°]), as well as constant distal tuning widths across the population of BVCs. In particular, the fixed distal tuning width was used because, unlike physical space, the precision of localisation in the concept space can depend only on the conceptual cues themselves rather than the location of boundaries. This contrasts 2D physical spaces, where the precision with which sensory observations of boundaries can be used to accurately guide self-localisation diminishes with distance from them^21^ - this is incorporated in the traditional BVC model by increasing BVC distal tuning widths with their preferred distal tuning to boundaries.^22^ Similar results are established when BVC distal tuning widths, σ_dis_, increase with distal tuning d_i_ as in Hartley et al.^17^ (BVC model LL = -4556.4 for σ_dis_= 200 + d_i_/50).

#### Alternative geometric models: Fixed ratio

We generated replacement predictions in the updated concept space that maintains a fixed ratio to the boundaries in the environment. Here, each location in each environment is parameterised by a vector of length 2: [R_NS_, R_EW_] where R_NS_ and R_EW_ are the ratios between the shortest distances to the North-South and East-West walls respectively. Displacement predictions were derived by first calculating the dissimilarity between object locations in the square to all locations in the rectangle using the L2 norm between the [R_NS_, R_EW_] vectors at the two locations. Dissimilarity scores were then turned into similarity scores by subtracting the dissimilarity from its maximum value across the rectangular environment.^17^ *Fixed distance*. We generated replacement predictions in the updated concept space that maintains a fixed distance to the two nearest boundaries in the environment.^17^ Here, each location in each environment is parameterised by a vector of length 2: [D_NS_, D_EW_] where D_NS_ and D_EW_ are the shortest distances to the North-South and East-West walls respectively. Displacement predictions were derived by first calculating the dissimilarity between object locations in the square to all locations in the rectangle using the L2 norm between the [D_NS_, D_EW_] vectors at the two locations. Dissimilarity scores were then turned into similarity scores by subtracting the dissimilarity from its maximum value across the rectangular environment.^17^

#### Model likelihood testing

The distribution of similarity scores S_xy_ for each model were converted into a probability distribution P_xy_ using a Softmax function P_XY_ = exp (−S_XY_ /*τ*)/Σ_*XY*_ exp (−S_XY_ /*τ*)(Figure 4). The inverse temperature parameter *τ* controls the sharpness of the distribution and was fit to maximise the log-likelihood of each model given the participant replacement data across all object locations using the fmincon function MATLAB 2022b. Model log-likelihoods were calculated using the natural logarithm log_e_(P_xy_).

## Supplementary Material

Supplemental information can be found online at https://doi.org/10.1016/j.cub.2024.09.083.

Supplemental Information

## Highlights

- We tested the influence of category boundary changes on human memory
- Memory of specific exemplars was influenced by the geometry of the conceptual space
- Category boundaries exert similar effects on memory as spatial boundaries
- A BVC population model of place cell activity predicted the observed distortions

## In brief

Theves et al. test whether category boundaries exert similar effects on human memory as environmental boundaries. They observe memory distortions that mimic the effects of environmental boundary changes on hippocampal place fields and spatial memory and explain these with a boundary vector cell model of place cell firing.
