# Supplementary material for: Category boundaries modulate memory in a place-cell-like manner
Source: Curr Biol. Author manuscript; Available in PMC 2026 Oct 8. (PMC7619516; doi:10.1016/j.cub.2024.09.083)
Supplement: Supplementary file 1 [file EMS219677-supplement-Supplemental_Information.pdf]

Current Biology, Volume *34*

Supplemental Information

Category boundaries modulate memory in a place-cell-like manner

Stephanie Theves, Theo A.J. Schäfer, Volker Reisner, William de Cothi, and Caswell Barry

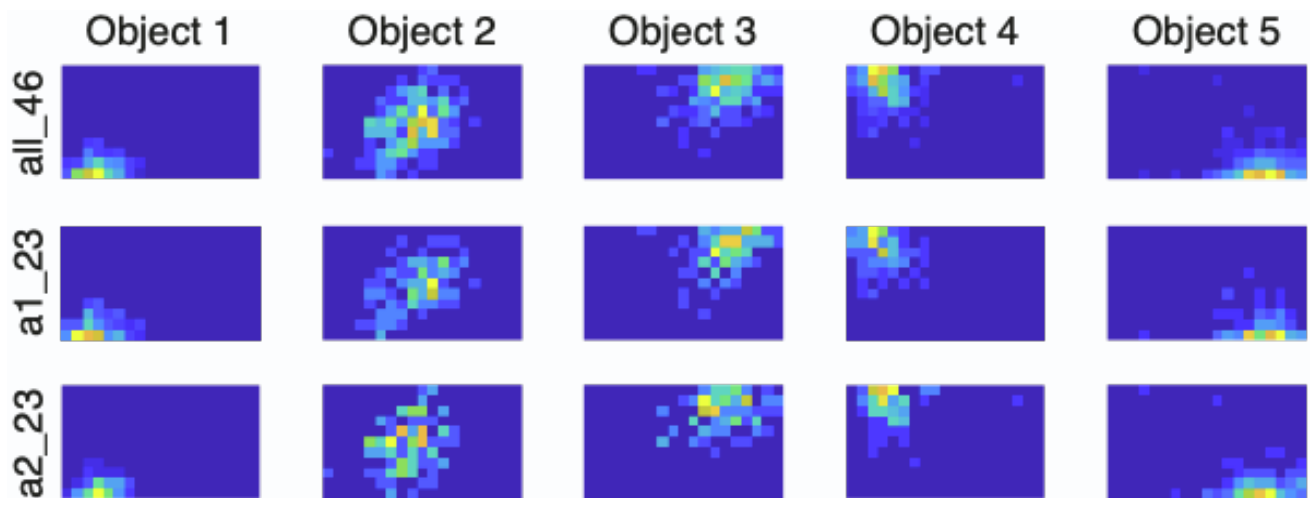

Figure S1 Response distributions in the memory test displayed per assignment group. Related to Figure 1. Response distributions displayed for all participants (N=46; first row) and separately for each feature (dot/stripe) to dimension (stretch/non-stretch) assignment group (a1:N=23; a2: N=23; middle & bottom row). Distribution a1 and a2 do not differ (t=0, p=1).

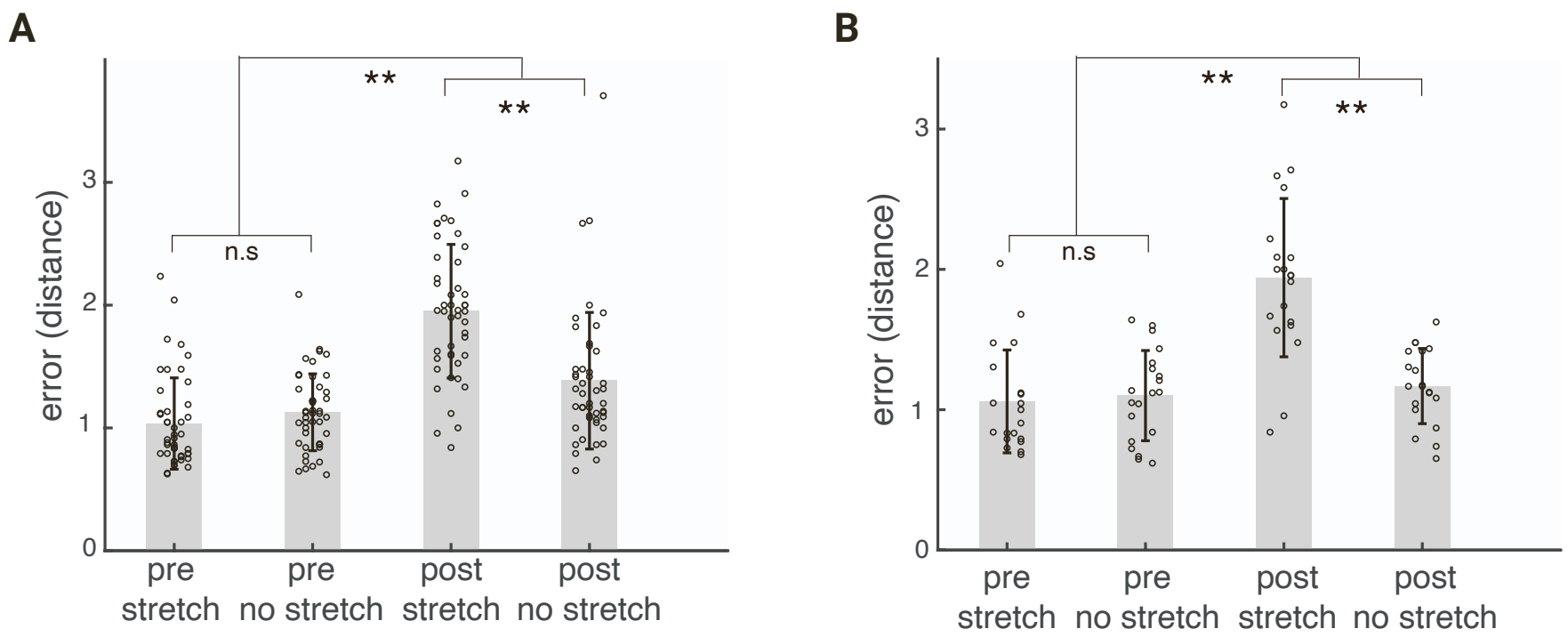

Figure S2 Replication of the time x dimension interaction on memory. Related to Figure 3. The effect is replicated in uncorrected trials only in A) all 46 participants and B) only participants that experienced <= 2 corrections in POST (cf. methods). A) We find a main effect of time (pre, post; F=12.29, p<.0006), a main effect of dimension (stretched, non-stretched; F=75 p=0), and a time x dimension interaction effect (F=23.65, p=0). Post-hoc t-tests reveal no significant memory differences between the stretched and the non-stretched dimension in the pre-test (t= -1.416, p=.164), but in the post-test (t=5.272, p<.0001; Bonferroni adjusted alpha level of .025). B) We find main effects of dimension (F(1, 76)=17, p<.00009), timepoint (F(1,76)=28, p<.0000008), and a time x dimension interaction effect (F(1,76)=21, p<.00001). Post-hoc t-tests reveal no significant memory differences between the stretched and the non-stretched dimension in the pre-test (t= -0.467, p=.646), but in the post-test (t=4.791, p<.0001).

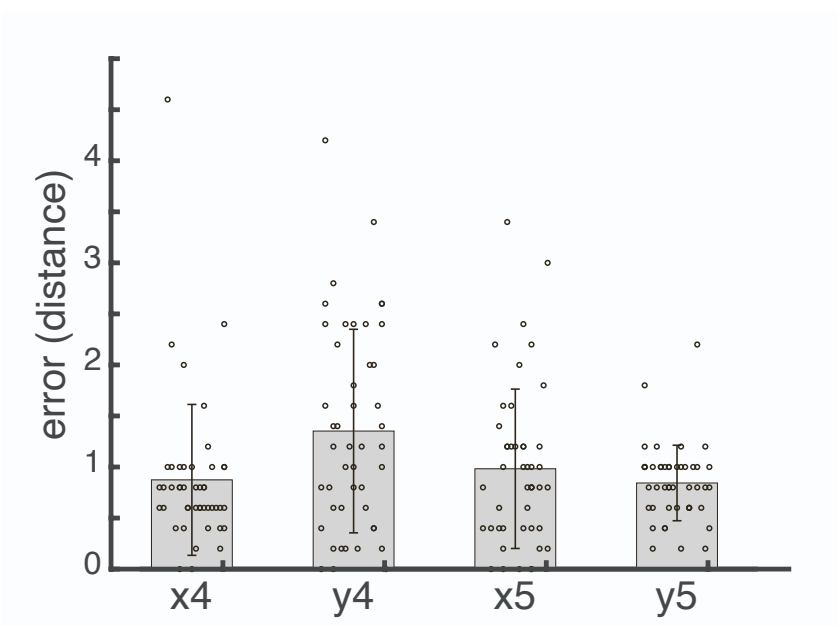

Figure S3 PRE-stretch position memory per dimension. Related to Figure 4. For positions 4 and 5, which both combine low/high feature values (upper left and lower right corner of the category space), pre-test memory is better for the dimension with the lower feature value (error y>x for position 4, t=-2.61, p=.01; error x>y for position 5, t=1.1, p=.27).

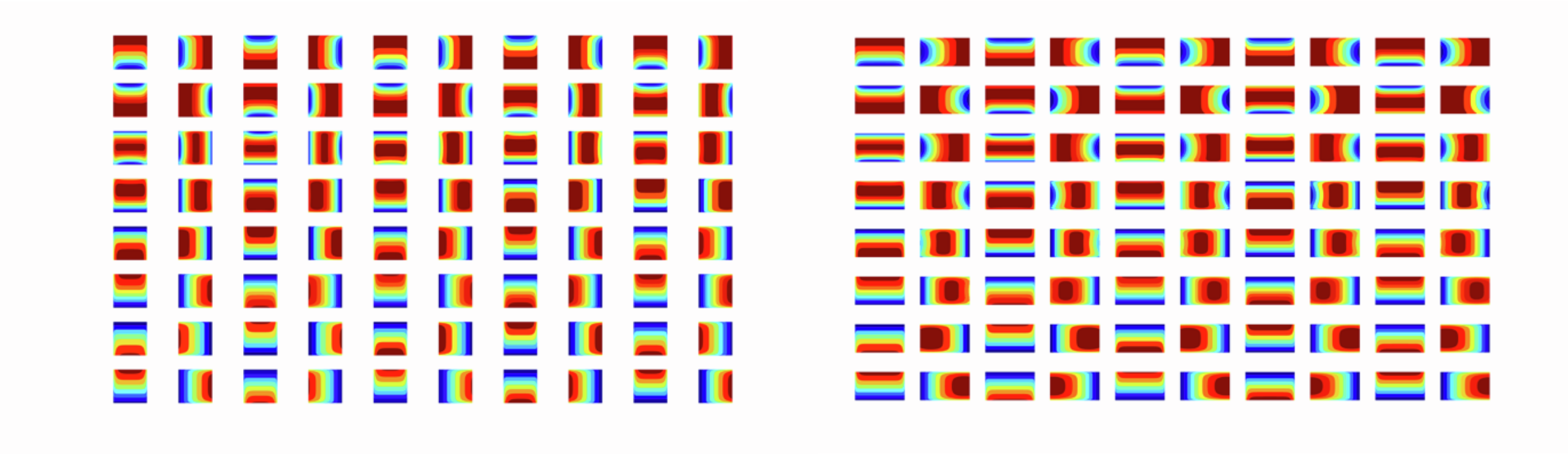

Figure S4 Rate maps of simulated BVCs. **Related to Figure 4.** Rate maps of simulated BVCs in the square (left) and rectangular (right) environment.
